# Role of Neddylation in Neurodegenerative Diseases

**DOI:** 10.3390/neurosci3040038

**Published:** 2022-09-27

**Authors:** Manoj Govindarajulu, Sindhu Ramesh, Tharanth Shankar, Murali Krishna Kora, Timothy Moore, Muralikrishnan Dhanasekaran

**Affiliations:** 1 Department of Drug Discovery and Development, Auburn University Harrison School of Pharmacy, Auburn, AL 36849, USA; 2 Department of Internal Medicine, Ramaiah Medical College and Hospital, Bengaluru 560054, Karnataka, India; 3 Sveta Clinic, Bengaluru 560038, Karnataka, India

**Keywords:** neddylation, Alzheimer’s disease, Parkinson’s disease, neurodegenerative diseases, NEDD8, misfolded proteins

## Abstract

Neurodegenerative diseases are characterized by progressive loss of neurons in specific regions of the brain. Neuronal death is often associated with the accumulation of misfolded proteins due to genetic mutations or abnormal protein homeostasis. An essential mechanism for regulating the clearance of misfolded proteins is neddylation, a post-translational modification closely related to ubiquitination. Neddylation is brought about by conjugating neural precursor cell-expressed developmentally downregulated protein 8 (NEDD8) to target substrates through a cascade of cellular events. Neddylation is crucial for many biological processes, and dysfunctional neddylation is implicated in several neurodegenerative diseases. This review discusses the current understanding of the role of neddylation pathways in neurodegenerative disorders and the emergence of neddylation signaling as a potential target for drug discovery and development in neurodegenerative diseases.

## 1. Introduction

Neurodegenerative diseases such as Alzheimer’s disease (AD) and Parkinson’s disease (PD) continue to be significant clinical concerns affecting around 1 billion individuals worldwide. They account for approximately 12% of all mortalities in the world [1]. These disorders are characterized by abnormal accumulation of misfolded proteins leading to progressive loss of neuronal structure and function [2]. Some shared pathological hallmarks of neurodegenerative diseases include oxidative stress, mitochondrial dysfunction, neuroinflammation, dysfunctional protein quality control, autophagy, and lysosomal dysregulation, which lead to neuronal death [3,4,5]. Neurodegenerative diseases share a range of molecular and cellular pathologies, including protein aggregation, mitochondrial dysfunction, glutamate toxicity, calcium load, proteolytic stress, oxidative stress, neuroinflammation, and aging, which contribute to neuronal death [6].

Recent studies indicate that the deranged cellular protein homeostasis is an important mechanism underlying the pathogenesis of neurodegenerative diseases like AD and PD [7]. Post-translational modifications (PTMs) play pivotal roles in protein quality control and homeostasis, and alterations in the PTMs can lead to many forms of human diseases, including neurodegenerative diseases. Post-translational modification (PTM) of proteins refers to the reversible or irreversible chemical modifications of proteins formed after translation. More than 300 PTMs have been identified, including acetylation, methylation, phosphorylation, glycosylation, and ubiquitination, among several others.

The ubiquitin superfamily includes around 17 members, including ubiquitin (Ub) and several ubiquitin-like proteins (UBLs) such as small ubiquitin-related modifier (SUMO) proteins, neuronal precursor cell-expressed developmentally downregulated protein 8 (NEDD8), interferon-stimulated gene 15 (ISG15), HLA-F-adjacent transcript 10 (FAT10), ubiquitin-fold modifier 1 (UFM1), ubiquitin-related modifier (URM1), and autophagy-related proteins 8 (ATG8) [8].

The role of ubiquitin and other UBLs, such as SUMO and ATG8, have been linked to the pathogenesis of neurodegenerative diseases [9,10]. Dysfunction of ubiquitination and other UBLs can promote the accumulation of un/misfolded proteins and can lead to neurodegeneration. Since neddylation is considered a ubuiquitination-like modification, its dysfunction predisposes to the pathogenesis of neurodegenerative diseases. However, the significance of other UBLs, specifically NEDD8, remains less explored. In this review, we first discuss the function and regulation of the neddylation pathway and focus on the role of neddylation in neurodegenerative diseases including AD and PD. 

## 2. Function and Regulation of Protein Neddylation

**Figure 1:**
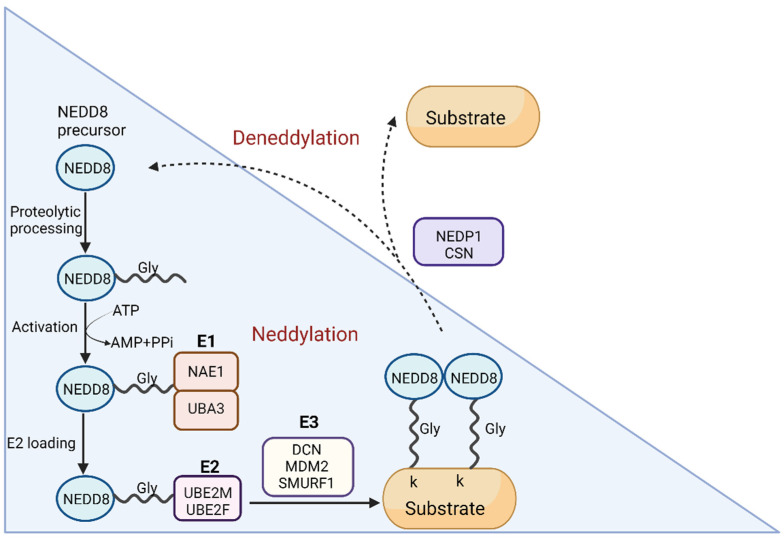
Proteolytic processing of precursor NEDD8 by DEN1/NEDP1 enzyme exposes C-terminal glycine (Gly76). Activation of mature NEDD8 occurs through ATP-dependent thioester linkage of Gly76 with cysteine at its active site and catalyzed by NAE, a heterodimer of UBA3 and NAE1 (APP-BP1). The active cysteine site of NAE then transfers activated NEDD8 to either E2 UBE2M or UBE2F’s active cysteine site. The C-terminal glycine of NEDD8 is exposed to nucleophilic assault by the substrate’s lysine residue due to E2′s interaction with an E3 ligase. This results in the covalent attachment of NEDD8 to the target proteins through an isopeptide bond. To remove NEDD8 moieties from substrates, the deneddylases NEDP1 and CSN catalyze the process. The Blue shaded region indicates neddylation. Abbreviations: APP-BP1—APP binding pro-tein 1; CSN—COP9 signalosome; DEN1—Human deneddylase 1; NEDD8—neural precursor cell expressed developmentally downregulated protein 8; NEDP1—NEDD8-specific protease; NAE—NEDD8-specific E1 activating enzyme; Uba3—NEDD8-activating enzyme E1 catalytic subunit. Image created in Biorender.com, accessed on 19 September 2022.

Neddylation is a ubiquitination-like protein (UBL) modification characterized by the addition of NEDD8 to lysine residue(s) of the substrate protein. The NEDD8 is ubiquitously expressed and evolutionarily shares 100% homology among mouse, rat, and human orthologs [11,12]. NEDD8 is initially synthesized as a precursor protein consisting of 81 amino acid residues, which subsequently is cleaved by the action of several proteases (UCH-L3, USP21, and NEDP1) to expose the carboxy-terminal glycine (Gly) 76 [13,14]. Similar to ubiquitination, the conjugation of NEDD8 to target proteins is mediated sequentially involving NEDD8-specific E1 activating enzymes (NAEs) and E2 conjugated enzymes (UBC12/UBE2M and UBE2F), and several unknown E3 ligases. The C-terminal residues of mature NEDD8 are activated ATP-dependent by forming a thioester bond with E1 enzymes (NAE). The activated NEDD8 is then transferred from the active cysteine site of NAE to the active cysteine site of E2 conjugating enzymes. Interaction of the E2 conjugating enzyme with E3 ligases leads to the transfer of the NEDD8 and the formation of an isopeptide bond between the C-terminal glycine-76 of NEDD8 and an ε-amino group of lysine in the target protein [15,16]. Neddylation is regulated and reversed by deneddylation, a process in which NEDD8 is removed from the protein substrates and is mediated by a group of deneddylase enzymes (Figure 1). Two important deneddylases that are NEDD8 specific and capable of removing conjugated NEDD8 from the neddylated substrate proteins include COP9 signalosome (CSN) and NEDD8-specific protease 1 (NEDP1). Several other deneddylases which target NEDD8 and ubiquitin include USP21, Ataxin-2, PfUCH54, UCH-L1, and UCH-L2 [17].

To date, several cullin and non-cullin proteins have been identified as physiological substrates for neddylation. Cullin proteins are molecular scaffolds which provide support for ubiquitin ligases (E3). They combine with RING proteins to form *Cullin-RING ubiquitin ligases (CRLs)* and play a pivotal role in post-translational modifications of cellular proteins involving ubiquitin [18]. The cullin family includes Cul-1, -2, -3, -4 A, -4B, -5, -7, and -9, each a scaffold subunit of Cullin-RING ligases (CRLs), and all cullins are modified by neddylation [19]. The CRLs consist of cullin proteins binding to an adaptor protein such as S-phase kinase-associated protein 1 (SKP1) in CRL1 and a substrate receptor protein (such as F-box protein in CRL1) at the N-terminus and a RING protein (RBX1 or RBX2) at the C-terminus [20,21]. The NEDD8 attaches to the C-terminal lysine residue of cullins and activates CRLs by causing a structural change in the CRLs to facilitate access of substrates for ubiquitylation [22]. While the neddylation of cullin family members is well characterized mechanistically, neddylation modification of non-cullin substrates is less studied. The key role of NEDD8 is to target cullin scaffold proteins, thereby increasing the activity of CRLs, which are mainly involved in the control of cell cycle and cellular proliferation [20,23]. Apart from conjugating CRLs, neddylation also mediates the enzymatic activity, transcriptional function, protein stability, and partner interaction of several non-cullin substrates [12]. 

## 3. Role of Neddylation in Neurodegenerative Diseases

As discussed earlier, neddylation is a ubiquitination-like modification that conjugates NEDD8 to its substrate proteins and regulates aspects of certain biological processes. Physiological levels of protein neddylation contribute to nerve growth, synapse strength, neurotransmission, and synaptic plasticity, whereas overactivation of protein neddylation pathways lead to apoptosis and neuronal death. Additionally, impaired neddylation causes neurodegenerative diseases indicating that different level of neddylation pathway contribute to the opposing disease progression. The following section discuss the current understanding of neddylation function in neurodegenerative diseases. 

### 3.1. Role of Neddylation in Alzheimer’s Disease

Alzheimer’s disease (AD) is the most common neurodegenerative disease characterized by progressive memory deficits, cognitive impairment, and in later stages, behavioral changes. The pathological hallmarks of the disease include extracellular senile plaques containing amyloid β-peptides and neurofibrillary tangles (NFTs) composed of hyperphosphorylated tau. In the initial stages, the abnormal proteins are degraded by either the ubiquitin-proteasome system (UPS) or the autophagy-driven vacuolar (lysosomal) proteolysis [24]. As the disease progresses, more insoluble proteins accumulate, causing the UPS machinery to fail due to excess protein deposition. Alternatively, conformational changes in protein substrates that prevent UPS degradation and recognition can also occur. NEDD8, a Ub-like protein, conjugates target proteins similarly to Ubiquitin and promotes the degradation of abnormal proteins. Under physiological conditions, NEDD8 is predominantly localized in the nucleus and functions to suppress DNA replication, cell cycle re-entry, and cell death [25]. However, in AD patients, neurons show decreased NEDD8 in the nuclei and its translocation to the cytoplasm [26,27]. Furthermore, NEDD8 colocalizes with ubiquitin and proteasome components in protein inclusions and is involved in the formation of ubiquitinated aggregates in the brain [28,29]. This is evident by the presence of NEDD8 immunoreactivity in ubiquitinated inclusions such as neurofibrillary tangles in AD indicating that neddylation dysregulation is characteristic of AD [29]. 

**Figure 2:**
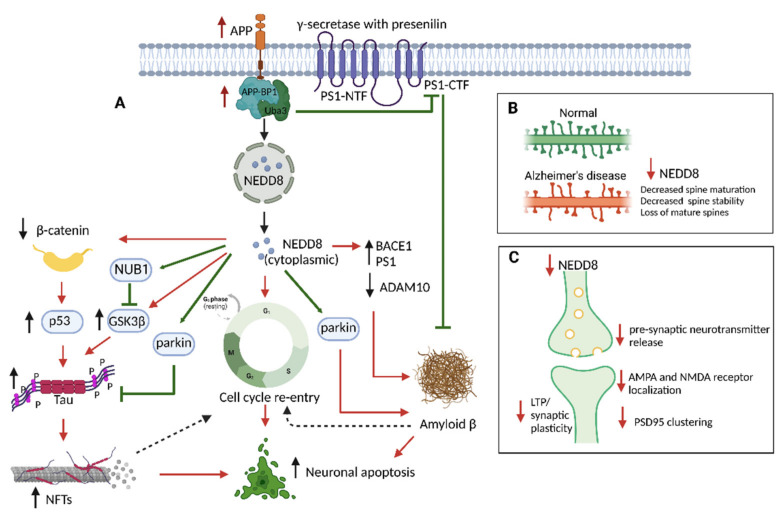
Role of neddylation in Alzheimer’s disease. (**A**) Increased expression of APP leads to upregulation of APP-BP1 and NEDD8 translocation from the nucleus to cytoplasm leading to cell cycle re-entry and apoptosis of neurons. The downstream effects of cytoplasmic NEDD8 include downregulation of β-catenin with an associated increase in p53, GSK3β, BACE1, and PS1 leading to tau hyperphosphorylation and Aβ. NEDD8 activates NUB1, which inhibits GSK3β and tau hyperphosphorylation. NEDD8 interacts with parkin and increases the ubiquitination of p-Tau and Aβ. Red arrows indicate detrimental and green arrows indicate a protective effect. (**B**) Downregulation of NEDD8 affects spine maturation, spine stability, and loss of mature spines in AD. (**C**) Downregulation of NEDD8 impairs the presynaptic and postsynaptic expression and function, leading to impaired synaptic neurotransmission. Abbreviations: Aβ—Amyloid beta; APP—Amyloid precursor protein; APP-BP1—APP binding protein 1; ADAM10—ADAM Metallopeptidase Domain 10; BACE1—beta-site amyloid precursor protein cleaving enzyme 1; GSK3β—Glycogen Synthase Kinase-3 Beta; LTP—Long term potentiation; NEDD8—neural precursor cell expressed developmentally downregulated protein 8; NFT—Neurofibrillary tangles; NUB1—NEDD8 ultimate buster 1; PS1—presenilin1; Uba3—NEDD8-activating enzyme E1 catalytic subunit; Image created in Biorender.com, accessed on 19 Septmber 2022.

The amyloid precursor protein (APP) is a transmembrane protein expressed predominantly in the neurons, and proteolytic cleavage of APP involving β- and γ-secretase enzymes generates the toxic amyloid beta (Aβ) [30]. Amyloid precursor protein binding protein-1 (APP-BP1) is one of the several proteins interacting with APP’s carboxy-terminal and serving as an activation enzyme for NEDD8. APP-BP1 is a bipartite enzyme consisting of NEDD8-activating enzyme E1 regulatory subunit 1 (NAE1) and NEDD8-activating enzyme E1 catalytic subunit (Uba3) [31]. In dividing cells, APP-BP1 expression drives the cell cycle through the S-M checkpoint. However, neurons are considered terminally differentiated (non-dividing and at the silent G0 phase) and are incapable of re-entering the cell cycle [32]. Abnormal cell cycle re-entry of neurons can induce apoptosis and neuronal death (Figure 2A) [33,34,35]. One of the mechanisms by which neurons re-enter the cell cycle is through the translocation of NEDD8 from the nucleus to the cytoplasm as discussed earlier. For instance, treatment with Aβ42 peptide in neurons increases APP-BP1 levels and translocation of NEDD8 from the nucleus to the cytoplasm promoting neuronal death. Cell cycle re-entry is considered a cause rather than a consequence of neurodegeneration and is suggested to be an early event in AD. Interestingly, phenotypic changes characteristic of cell cycle re-entry has been noted in degenerating AD neurons [36,37,38]. Furthermore, the increased cell cycle markers in AD brains are regulated by the APP-BP1 pathway, indicating that APP-BP1 is an upstream regulator of cell cycle markers that go dysfunctional in AD [39]. 

Another characteristic feature of cell cycle re-entry and neuronal apoptosis mediated by APP-BP1 is the downregulation of β-catenin and increased p53 activation (Figure 2A). In the brain, Wnt/β-catenin signaling is important in regulating neuronal survival and neurogenesis, synaptic plasticity and blood–brain barrier integrity [40]. Since, the physiological function of APP is to downregulate the levels of β-catenin, decreased levels of β-catenin inhibits Wnt/Wingless signal transduction pathways related to neurogenesis, thereby causing neuronal apoptosis. In patients with familial AD, mutations in APP lead to abnormally amplified APP function resulting in hyperactivation of neddylation and the associated reduction in β-catenin below the threshold levels [41]. In primary neurons expressing APP and in familial AD mutants of APP, increased APP expression promotes β-catenin degradation leading to loss of cell–cell adhesion and making neurons vulnerable to apoptosis [41,42]. Hence, APP-BP1 interaction with APP signals neurons to divide via the neddylation pathway, causing apoptosis. Supporting this view, a mouse model of AD (Tg2576) overexpressing a mutant form of APP showed increased protein expression of APP-BP1. Furthermore, increased protein levels of APP-BP1 in lipid rafts are noted in primary neuronal cultures overexpressing APP and in the human AD hippocampus. Additionally, the increase in APP-BP1 levels in lipid rafts is associated with the translocation of NEDD8 from the nucleus to cytoplasm in APP overexpressing neuronal cultures and AD hippocampal neurons [26]. Hence, neuronal death induced by overexpression of APP is mediated by the interaction of APP with APP-BP1 in the lipid rafts, which activates the neddylation pathway and thereby inducing cycle entry. 

Parkin is an E3 ubiquitin ligase that regulates cellular homeostasis, including ubiquitination of unwanted proteins and cellular entities [43]. Parkin-mediated ubiquitination of un/misfolded proteins is attributed to phosphorylated ubiquitin (p-Ub) binding to parkin [44]. Typically, the parkin is kept at an auto-inhibitory state by binding to the RING2 (a fascinating new gene) domain. Binding of p-Ub to parkin results in detachment of RING2, thereby exposing Ser65 residue of parkin for phosphorylation [45,46]. The phosphorylation of parkin Ser65 is mediated by PTEN-induced putative kinase protein 1 (PINK1). Similar to p-Ub, NEDD8 binds to and phosphorylates Ser65 of parkin, suggesting that either p-Ub or NEDD8 mediated activation of parkin leads to parkin-mediated ubiquitination of un/misfolded proteins. Hence, decreased levels of PINK1 in the brains of AD patients have been suggested to account, at least partly, for failures in ubiquitination in AD [47]. Under conditions of neuronal stress as seen in AD, NEDD8 cytoplasmic translocation occurs alongside elevated parkin, and parkin-NEDD8 interaction characterized by increased colocalization of cytoplasmic NEDD8 with parkin is noted [48]. Similar to AD brains, IL-1β-treated rat primary neuron cultures showed translocation of NEDD8 from nucleus to cytoplasm, indicating that neuroinflammation facilitates parkin activation and drives NEDD8 translocation from nucleus to cytoplasm to bind with parkin and promote clearance of unwanted proteins and aggregates [48]. Despite an increase in NEDD8-related parkin activation to clear the aggregates in AD occurs, this mechanism is still not sufficient to remove the aggregates (Figure 2A). 

One of the hallmarks of AD pathogenesis is Aβ aggregation and deposition in the extracellular space in the brain. In the amyloidogenic pathway, the APP is sequentially cleaved first by β-secretase followed by γ–secretase to generate the toxic Aβ. The γ–secretase is a multi-protein complex consisting of presenilin (PS) as the primary catalytic constituent [49]. APP-BP1 interacts with PS1 and decreases Aβ42 levels by facilitating PS1-CTF degradation (Figure 2A). Furthermore, in primary neurons, suppression of APP-BP1 by siRNA resulted in increased intracellular Aβ42 [50]. Since the critical role of APP-BP1 is to neddylation cullins and activate CRLs, APP-BP1 knockdown induces stabilization of PS1, thereby promoting increased Aβ levels in the neurons [50]. Another important function of APP-BP1 is activating parkin-containing E3 ligase, which polyubiquitinates misfolded proteins through a Lys-63-linked ubiquitin chain [51,52]. Studies indicate that parkin expression ubiquitinates intracellular Aβ and promotes Aβ clearance through proteasomal degradation and activation of autophagy [53,54,55]. Selective inhibition of NAE and the NEDD8 pathway decreased β-Secretase 1 (BACE1) and PS1 levels along with upregulation of ADAM10 (α-secretase), indicating that NEDD8 modulates the regulation of APP proteolysis as seen in AD [56]. 

The potential role of neddylation dysregulation in tau pathology can be explained by the presence of anti-NEDD8 staining in the neurofibrillary tangles of AD patients [29]. Since increased cytoplasmic NEDD8 is noted in AD neurons, the possibility of excess NEDD8 being erroneously incorporated into the ubiquitin chains cannot be ruled out as tau undergoes polyubiquitination. A proteasome-associated protein, NEDD8 ultimate buster 1 (NUB1), is a NEDD8-interacting protein that negatively regulates NEDD8 by subjecting NEDD8 to proteasome-mediated degradation [57]. In addition, NUB1 and its isoform NUB1L facilitate proteasomal degradation of pathological proteins such as tau, synuclein, and huntingtin. In AD, pathological hyperphosphorylation of tau is mediated by glycogen synthase kinase 3β (GSK3β), and NUB1 has been shown to inhibit GSK3β-mediated hyperphosphorylation of tau by inducing GSK3β degradation (Figure 2A) [58]. Interestingly, NUB1 increases p62, autophagosomes, and lysosomal aggregation under compromised ubiquitin-proteasome function. Hence, under proteasome inhibition, NUB1 facilitates the clearance of aggregated tau by regulating the autophagy-lysosomal pathway [59].

Synaptic neurotransmission mediated by pre- and postsynaptic proteins is regulated by several post-translational modifications [60]. Specifically, the neddylation of several pre- and postsynaptic proteins are vital for maintaining spine maturation, spine stability, and synaptic function/transmission [61,62]. In primary neuronal culture, functional downregulation of NEDD8 by either shRNA against NEDD8, overexpression of dominant negative Ubc12 (Ubc12-C111S) that sequesters NEDD8, or treatment with the NAE inhibitor MLN-4924 prevented dendritic spine maturation. Additionally, in Nae1 conditional knockout mice, inactivation of the neddylation pathway resulted in reduced dendritic spine stability causing loss of excitatory synapses, impaired neurotransmission, and cognitive deficits in adult excitatory forebrain neurons (Figure 2B) [61]. The metabotropic glutamate 7 (mGlu7) receptor is primarily localized in the presynaptic active zone, where it suppresses the release of neurotransmitters by acting as an autoinhibitory receptor [63,64,65]. Studies indicate that post-translational modifications, including neddylation, regulate the stable neuronal surface expression of mGLu7. The mGlu7 is a target substrate for NEDD8 conjugation, and neddylation is essential for the localization of mGlu7 in the presynaptic active zone and the maturation of excitatory presynaptic terminals. 

Regarding postsynaptic proteins, postsynaptic density protein 95 (PSD-95) is a critical postsynaptic scaffolding protein present in the excitatory neurons and is a substrate for neddylation [61]. Neddylation of PSD95 is crucial for spine stability, spine maturation, and synaptic transmission. Inhibition of neddylation inhibited the development of mature dendritic spines. Mice carrying a specific ablation of neddylation in forebrain excitatory neurons exhibited synaptic instability, impaired neurotransmission, and cognitive deterioration. The expression level of PSD-95 is not affected by neddylation; instead, the diffusion of PSD-95 away from mature spines reduces PSD-95 clusters. This reduction in clustering is accompanied by a decrease in the frequency of miniature excitatory postsynaptic currents (mEPSCs), leading to deficits in synaptic transmission (Figure 2C) [61]. 

In addition to the morphological changes, neddylation can modulate the functional changes in the synapse. At the presynaptic level, the neddylation of presynaptic proteins regulates presynaptic neurotransmitter release by modulating vesicular release probability. Postsynaptically, neddylation is involved in the localization of AMPA and NMDA receptors, and inhibition of neddylation with NAE1 inhibitor MLN-4924 reduced the number of AMPA and NMDA receptors in the postsynaptic region (Figure 2C). Interestingly, inhibiting neddylation before the induction of long-term potentiation (LTP) in hippocampal slices reduced the expression of LTP, whereas inhibiting neddylation after the induction of LTP had no effect. These findings indicate that the neddylation of synaptic proteins is important for regulating basal synaptic transmission and synaptic plasticity [66]. 

Several lines of evidence indicate that synaptic dysfunction and synaptic plasticity deficits are hallmarks of AD pathogenesis. Interestingly neddylation of several essential synaptic proteins has been linked to synaptic dysfunction. Synaptic insulin resistance is one of the hallmarks of cognitive decline in metabolic syndrome [67,68]. Additionally, amyloid plaques with associated chronic inflammation and insulin resistance increase the risk of developing AD [69,70]. The induction of synaptic IR is linked to insulin-receptor substrate 1 (IRS1) degradation. A recent study by Confettura et al. demonstrated neddylation and subsequent downregulation of insulin-receptor substrate as a common factor linking amyloid plaques, neuroinflammation, and synaptic IR to cognitive decline. Neddylation-dependent IRS1 degradation involves cullins, specifically Cullin-7, that are highly expressed in the synaptic sites. Furthermore, the study demonstrated pharmacological inhibition of neddylation rescued synaptic plasticity deficits and memory impairment in a mouse model of high-risk aging [71]. Since the current line of evidence show a contradictory multifaceted role of neddylation in AD and AD pathogenesis by itself is complex, more studies need to be evaluated to determine the function of neddylation in AD to develop better therapeutic avenues. 

### 3.2. Role of Neddylation in Parkinson’s Disease

Parkinson’s disease is the second most common neurodegenerative disease characterized by loss of dopaminergic neurons in the substantia nigra and aggregation of intracellular α-synuclein. The familial form of PD represents 5–10% of cases, and the most common genes implicated in familial PD include Parkin, LRRK2, PINK1, alpha-synuclein, UCH-L1, and ATP13A2. Sporadic PD accounts for a majority of PD patients, and the combination of genetic and environmental risk factors plays a pivotal role in determining the onset of PD [72]. 

Among several genes, PTEN-induced kinase 1 (PINK1) and RING-between-RING (RBR) E3 ubiquitin-protein ligase (PRKN) genes are essential in mitochondrial quality control (mitophagy) and promote the degradation of several unfolded proteins via the ubiquitin proteasomal pathway as described earlier [73]. Hence, pathogenic mutants of PINK1 and parkin show reduced ubiquitin E3 ligase activity of parkin, thereby promoting the accumulation of mis/unfolded proteins [73]. Interestingly, both PINK1 and parkin undergo neddylation, and neddylation modification results in increased ubiquitin E3 ligase activity of parkin and stabilization of PINK1 fragment. Additionally, NEDD8 causes an increase in parkin activity through enhanced binding affinity for ubiquitin-conjugating E2 enzyme and the formation of complexes containing parkin and substrates [74]. Hence, reduced neddylation results in reduced ubiquitin E3 ligase activity and accumulated mis/unfolded proteins. Several studies indicate that impaired post-translational NEDD8 modification of PINK1 and parkin is associated with the pathogenesis of PD [44,74]. Furthermore, the neddylation of PINK1 and parkin are inhibited by PD neurotoxin MPP+ [44]. Lewy bodies (LBs), the histological hallmark of PD consisting of fibrillar aggregates of abnormal protein α-synuclein stained with anti-NEDD8 antibody, show NEDD8 immunoreactivity in pigmented midbrain dopaminergic neurons in PD patients [44]. Similarly, specific immunoreactivity to NEDD8 is detected in Lewy bodies in PD, indicating that neddylation is involved in PD pathogenesis [28,29]. These results suggest that parkin and PINK1 are regulated by neddylation and that impaired NEDD8 modification of these proteins likely contributes to PD pathogenesis. 

As indicated earlier, NUB1 negatively regulates the levels of NEDD8, and NUB1 likely contributes to the pathogenesis of PD and other synucleopathies, such as LB dementia and multiple system atrophy. A study by Tanji et al. showed that NUB1 inhibited the formation of Lewy Body-like inclusions consisting of α-synuclein, and an abnormally phosphorylated NUB1 termed p-NUB46 is seen in PD and Lewy Body dementia [75,76]. These results indicate that neddylation dysfunction of these proteins could contribute to the pathogenesis of PD. 

### 3.3. Role of Neddylation in Multiple Sclerosis

Multiple sclerosis (MS) is an autoimmune condition of the CNS that affects 2 million people worldwide [77]. It is characterized by demyelination and neurodegeneration, which results in persistent clinical impairment [78]. While it is evident that both hereditary and environmental influences have a role in the pathophysiology of multiple sclerosis, the exact cause of the disease is still unknown. Current immunomodulatory medications are only partially successful as MS progression is predominantly driven by immune-independent mechanisms and these medications only help in the early stages when there is still a component of inflammation [79]. Clinical trials conducted by Czuczman et al. in lymphoproliferative disorders have shown favorable findings by inhibiting neddylation using a small molecule inhibitor first-in-class inhibitor of NAE (pevonedistat) [80]. The relevance of neddylation in immune cell function in general, and T-cell role in particular, has not been investigated in multiple sclerosis, despite prior research finding a connection between the UPS activity and the regulation of myelin protein degradation and IFN-beta-1b therapy [81,82,83]. A recent transcriptomic study revealed the upregulation of genes associated with neddylation in T cells from people with MS [84]. The researchers discovered that inhibition of the NEDD8 activating enzyme using the specific inhibitor pevonedistat (MLN4924) significantly ameliorated disease severity in a mouse MS model by reducing inflammation through suppression of NF-κB. When paired with human RNA-seq data, these results suggest that the neddylation pathway is likely involved in the etiology of human multiple sclerosis [84]. However, there are hardly any studies to establish the molecular signaling pathways between neddylation and multiple sclerosis; further studies need to be performed to identify the importance of the neddylation pathway in multiple sclerosis. 

### 3.4. Role of Neddylation in Other Neurodegenerative Diseases

Huntington’s disease (HD) is an inherited monogenic autosomal dominant neurodegenerative disorder caused by a toxic gain of function of the huntingtin gene (HTT). The expanded CAG repeat in the HTT gene forms insoluble aggregates in the neurons leading to neuronal death [85]. However, the specific mechanisms of how misfolded/mutated HTT (mHTT) leads to disease pathogenesis remain unclear. The protein NUB1 interacts with N-terminal fragments of mHTT protein and downregulates mHTT expression by proteasomal degradation both in in vitro and in vivo [86,87,88]. The NUB1-mediated mHTT clearance requires NEDD8 and CUL3, a NEDD8 substrate [88]. Since increasing the levels of CUL3 leads to mHTT clearance and it is known that activation of cullin E3 ligases is dependent on neddylation, targeting neddylation of CUL3 might offer a therapeutic avenue. Alternatively, inducing NUB1 by treatment with interferon-β decreased mHTT and rescued neuronal toxicity indicating NUB1 as a possible therapeutic target in Huntington’s disease [88]. 

Fragile X syndrome is another neurodegenerative disorder caused by expansion of CGG repeats in the fragile X mental retardation 1 (FMR1) gene and characterized by intentional tremors, ataxia, behavioral deficits, and cognitive impairments. In *Drosophila* model of Fragile X syndrome, reduced neddylation levels resulted in enhanced neurodegeneration phenotypes, and activation of neddylation prevented neurotoxicity caused by CGG repeats. The impaired neddylation activity leads to decreased E3 ligase activity of neddylation substrates, Cul3 and Vhl, resulting in increased expression of Sima (*Drosophilia* HIF1α) [89]. Hence, modulating the neddylation pathway could be a potential therapeutic target in Fragile X syndrome though more mechanistic studies need to be performed. 

Spinocerebellar ataxia type 3 (SCA3) or Machado–Joseph disease (MJD) is an autosomal dominant condition characterized by progressive cerebellar ataxia and other neurological manifestations. The disease is due to the expansion of CAG repeats in the coding region of the *ATXN3* gene [90]. The disease is characterized by the presence of nuclear and cytoplasmic inclusions, which are ubiquitylated. Interestingly, the presence of NEDD8 in ubiquitin-reactive neuronal and glial inclusions was noted in SCA3 [29]. A study by Ferro et al. demonstrated a direct interaction between ATXN3 and NEDD8 through its Josephin domain (JD) and not dependent on the ubiquitin-interacting motifs. However, the functional consequences of the interaction need further investigation [91]. 

## 4. Conclusions

Neddylation of proteins has emerged as an essential post-translation modification in the brain and other tissues. The regulation of neddylation varies under different physiological and/or pathological conditions. This study provides valuable insights into the dysregulation of neddylation in several major neurodegenerative diseases. Further research needs to be performed to discover unknown details about gene regulation related to neurodegenerative diseases and neddylation as a critical mechanism in the etiology of the disease, with the hope of discovering specific disease-modifying drugs. Neddylation may, therefore, be a new potential therapeutic target for neurodegenerative diseases and agreeable to being used in combination with other disease-modifying agents.

## Data Availability

Not applicable.

## References

[1] Feigin V.L., Vos T., Nichols E., Owolabi M.O., Carroll W.M., Dichgans M., Deuschl G., Parmar P., Brainin M., Murray C. (2020). The global burden of neurological disorders: Translating evidence into policy. Lancet Neurol..

[2] Sweeney P., Park H., Baumann M., Dunlop J., Frydman J., Kopito R., McCampbell A., Leblanc G., Venkateswaran A., Nurmi A. (2017). Protein misfolding in neurodegenerative diseases: Implications and strategies. Transl. Neurodegener..

[3] Kwon H.S., Koh S.-H. (2020). Neuroinflammation in neurodegenerative disorders: The roles of microglia and astrocytes. Transl. Neurodegener..

[4] Ramesh S., Govindarajulu M., Jones E., Suppiramaniam V., Moore T., Dhanasekaran M. (2018). Mitochondrial dysfunction and the role of Mitophagy in Alzheimer’s disease. Alzheimer’s Disease & Treatment.

[5] Le Guerroué F., Youle R.J. (2021). Ubiquitin signaling in neurodegenerative diseases: An autophagy and proteasome perspective. Cell Death Differ..

[6] Gan L., Cookson M.R., Petrucelli L., La Spada A.R. (2018). Converging pathways in neurodegeneration, from genetics to mechanisms. Nat. Neurosci..

[7] Höhn A., Tramutola A., Cascella R. (2020). Proteostasis Failure in Neurodegenerative Diseases: Focus on Oxidative Stress. Oxid. Med. Cell. Longev..

[8] Bedford L., Lowe J., Dick L.R., Mayer R.J., Brownell J.E. (2011). Ubiquitin-like protein conjugation and the ubiquitin-proteasome system as drug targets. Nat. Rev. Drug Discov..

[9] Kawabe H., Brose N. (2011). The role of ubiquitylation in nerve cell development. Nat. Rev. Neurosci..

[10] Yang Y., Kim A.H., Bonni A. (2010). The dynamic ubiquitin ligase duo: Cdh1-APC and Cdc20-APC regulate neuronal morphogenesis and connectivity. Curr. Opin. Neurobiol..

[11] Kamitani T., Kito K., Nguyen H.P., Yeh E.T. (1997). Characterization of NEDD8, a developmentally down-regulated ubiquitin-like protein. J. Biol. Chem..

[12] Xirodimas D.P. (2008). Novel substrates and functions for the ubiquitin-like molecule NEDD8. Biochem. Soc. Trans..

[13] Gan-Erdene T., Nagamalleswari K., Yin L., Wu K., Pan Z.Q., Wilkinson K.D. (2003). Identification and characterization of DEN1, a deneddylase of the ULP family. J. Biol. Chem..

[14] Kandala S., Kim I.M., Su H. (2014). Neddylation and deneddylation in cardiac biology. Am. J. Cardiovasc. Dis..

[15] Gong L., Yeh E.T. (1999). Identification of the activating and conjugating enzymes of the NEDD8 conjugation pathway. J. Biol. Chem..

[16] Soucy T.A., Smith P.G., Milhollen M.A., Berger A.J., Gavin J.M., Adhikari S., Brownell J.E., Burke K.E., Cardin D.P., Critchley S. (2009). An inhibitor of NEDD8-activating enzyme as a new approach to treat cancer. Nature.

[17] Zhao Y., Morgan M.A., Sun Y. (2014). Targeting Neddylation pathways to inactivate cullin-RING ligases for anticancer therapy. Antioxid. Redox Signal..

[18] Sarikas A., Hartmann T., Pan Z.-Q. (2011). The cullin protein family. Genome Biol..

[19] Zhou L., Zhang W., Sun Y., Jia L. (2018). Protein neddylation and its alterations in human cancers for targeted therapy. Cell. Signal..

[20] Petroski M.D., Deshaies R.J. (2005). Function and regulation of cullin-RING ubiquitin ligases. Nat. Rev. Mol. Cell Biol..

[21] Deshaies R.J. (1999). SCF and Cullin/Ring H2-based ubiquitin ligases. Annu. Rev. Cell Dev. Biol..

[22] Duda D.M., Borg L.A., Scott D.C., Hunt H.W., Hammel M., Schulman B.A. (2008). Structural insights into NEDD8 activation of cullin-RING ligases: Conformational control of conjugation. Cell.

[23] Hori T., Osaka F., Chiba T., Miyamoto C., Okabayashi K., Shimbara N., Kato S., Tanaka K. (1999). Covalent modification of all members of human cullin family proteins by NEDD8. Oncogene.

[24] Tyedmers J., Mogk A., Bukau B. (2010). Cellular strategies for controlling protein aggregation. Nat. Rev. Mol. Cell Biol..

[25] Tateishi K., Omata M., Tanaka K., Chiba T. (2001). The NEDD8 system is essential for cell cycle progression and morphogenetic pathway in mice. J. Cell Biol..

[26] Chen Y., Liu W., McPhie D.L., Hassinger L., Neve R.L. (2003). APP-BP1 mediates APP-induced apoptosis and DNA synthesis and is increased in Alzheimer’s disease brain. J. Cell Biol..

[27] Chen Y., Neve R.L., Liu H. (2012). Neddylation dysfunction in Alzheimer’s disease. J. Cell Mol. Med..

[28] Dil Kuazi A., Kito K., Abe Y., Shin R.W., Kamitani T., Ueda N. (2003). NEDD8 protein is involved in ubiquitinated inclusion bodies. J. Pathol..

[29] Mori F., Nishie M., Piao Y.S., Kito K., Kamitani T., Takahashi H., Wakabayashi K. (2005). Accumulation of NEDD8 in neuronal and glial inclusions of neurodegenerative disorders. Neuropathol. Appl. Neurobiol..

[30] Zheng H., Koo E.H. (2011). Biology and pathophysiology of the amyloid precursor protein. Mol. Neurodegener..

[31] Walden H., Podgorski M.S., Huang D.T., Miller D.W., Howard R.J., Minor D.L., Holton J.M., Schulman B.A. (2003). The structure of the APPBP1-UBA3-NEDD8-ATP complex reveals the basis for selective ubiquitin-like protein activation by an E1. Mol. Cell.

[32] McShea A., Wahl A.F., Smith M.A. (1999). Re-entry into the cell cycle: A mechanism for neurodegeneration in Alzheimer disease. Med. Hypotheses.

[33] Nagy Z., Esiri M.M., Cato A.M., Smith A.D. (1997). Cell cycle markers in the hippocampus in Alzheimer’s disease. Acta Neuropathol..

[34] Neve R.L., McPhie D.L. (2007). Dysfunction of amyloid precursor protein signaling in neurons leads to DNA synthesis and apoptosis. Biochim. Biophys. Acta.

[35] Yang Y., Geldmacher D.S., Herrup K. (2001). DNA replication precedes neuronal cell death in Alzheimer’s disease. J. Neurosci..

[36] Barrio-Alonso E., Hernández-Vivanco A., Walton C.C., Perea G., Frade J.M. (2018). Cell cycle reentry triggers hyperploidization and synaptic dysfunction followed by delayed cell death in differentiated cortical neurons. Sci. Rep..

[37] Lee H.G., Casadesus G., Zhu X., Castellani R.J., McShea A., Perry G., Petersen R.B., Bajic V., Smith M.A. (2009). Cell cycle re-entry mediated neurodegeneration and its treatment role in the pathogenesis of Alzheimer’s disease. Neurochem. Int..

[38] Bonda D.J., Lee H.P., Kudo W., Zhu X., Smith M.A., Lee H.G. (2010). Pathological implications of cell cycle re-entry in Alzheimer disease. Expert Rev. Mol. Med..

[39] Chen Y., Neve R., Zheng H., Griffin W., Barger S., Mrak R. (2014). Cycle on Wheels: Is APP Key to the AppBp1 Pathway?. Austin Alzheimers Parkinsons Dis..

[40] Jia L., Piña-Crespo J., Li Y. (2019). Restoring Wnt/β-catenin signaling is a promising therapeutic strategy for Alzheimer’s disease. Mol. Brain.

[41] Chen Y.Z. (2004). APP induces neuronal apoptosis through APP-BP1-mediated downregulation of beta-catenin. Apoptosis.

[42] Chen Y., Bodles A.M. (2007). Amyloid precursor protein modulates β-catenin degradation. J. Neuroinflamm..

[43] Zhang C.W., Hang L., Yao T.P., Lim K.L. (2015). Parkin Regulation and Neurodegenerative Disorders. Front. Aging Neurosci..

[44] Choo Y.S., Vogler G., Wang D., Kalvakuri S., Iliuk A., Tao W.A., Bodmer R., Zhang Z. (2012). Regulation of parkin and PINK1 by neddylation. Hum. Mol. Genet..

[45] McWilliams T.G., Barini E., Pohjolan-Pirhonen R., Brooks S.P., Singh F., Burel S., Balk K., Kumar A., Montava-Garriga L., Prescott A.R. (2018). Phosphorylation of Parkin at serine 65 is essential for its activation in vivo. Open Biol..

[46] Koyano F., Okatsu K., Kosako H., Tamura Y., Go E., Kimura M., Kimura Y., Tsuchiya H., Yoshihara H., Hirokawa T. (2014). Ubiquitin is phosphorylated by PINK1 to activate parkin. Nature.

[47] Du F., Yu Q., Yan S., Hu G., Lue L.F., Walker D.G., Wu L., Yan S.F., Tieu K., Yan S.S. (2017). PINK1 signalling rescues amyloid pathology and mitochondrial dysfunction in Alzheimer’s disease. Brain.

[48] Balasubramaniam M., Parcon P.A., Bose C., Liu L., Jones R.A., Farlow M.R., Mrak R.E., Barger S.W., Griffin W.S.T. (2019). Interleukin-1β drives NEDD8 nuclear-to-cytoplasmic translocation, fostering parkin activation via NEDD8 binding to the P-ubiquitin activating site. J. Neuroinflamm..

[49] Hampel H., Hardy J., Blennow K., Chen C., Perry G., Kim S.H., Villemagne V.L., Aisen P., Vendruscolo M., Iwatsubo T. (2021). The Amyloid-β Pathway in Alzheimer’s Disease. Mol. Psychiatry.

[50] Chen Y., Bodles A.M., McPhie D.L., Neve R.L., Mrak R.E., Griffin W.S. (2007). APP-BP1 inhibits Abeta42 levels by interacting with Presenilin-1. Mol. Neurodegener..

[51] Olzmann J.A., Li L., Chudaev M.V., Chen J., Perez F.A., Palmiter R.D., Chin L.S. (2007). Parkin-mediated K63-linked polyubiquitination targets misfolded DJ-1 to aggresomes via binding to HDAC6. J. Cell Biol..

[52] Imai Y., Soda M., Takahashi R. (2000). Parkin suppresses unfolded protein stress-induced cell death through its E3 ubiquitin-protein ligase activity. J. Biol. Chem..

[53] Burns M.P., Zhang L., Rebeck G.W., Querfurth H.W., Moussa C.E. (2009). Parkin promotes intracellular Abeta1-42 clearance. Hum. Mol. Genet..

[54] Rosen K.M., Moussa C.E., Lee H.K., Kumar P., Kitada T., Qin G., Fu Q., Querfurth H.W. (2010). Parkin reverses intracellular beta-amyloid accumulation and its negative effects on proteasome function. J. Neurosci. Res..

[55] Khandelwal P.J., Herman A.M., Hoe H.S., Rebeck G.W., Moussa C.E. (2011). Parkin mediates beclin-dependent autophagic clearance of defective mitochondria and ubiquitinated Abeta in AD models. Hum. Mol. Genet..

[56] Shukla M., Chinchalongporn V., Govitrapong P. (2020). Melatonin Prevents Neddylation Dysfunction in Aβ42-Exposed SH-SY5Y Neuroblastoma Cells by Regulating the Amyloid Precursor Protein- Binding Protein 1 Pathway. Curr. Alzheimer Res..

[57] Hosono T., Tanaka T., Tanji K., Nakatani T., Kamitani T. (2010). NUB1, an interferon-inducible protein, mediates anti-proliferative actions and apoptosis in renal cell carcinoma cells through cell-cycle regulation. Br. J. Cancer.

[58] Richet E., Pooler A.M., Rodriguez T., Novoselov S.S., Schmidtke G., Groettrup M., Hanger D.P., Cheetham M.E., van der Spuy J. (2012). NUB1 modulation of GSK3β reduces tau aggregation. Hum. Mol. Genet..

[59] Guarascio R., Salih D., Yasvoina M., Edwards F.A., Cheetham M.E., van der Spuy J. (2020). Negative Regulator of Ubiquitin-Like Protein 1 modulates the autophagy-lysosomal pathway via p62 to facilitate the extracellular release of tau following proteasome impairment. Hum. Mol. Genet..

[60] Yi J.J., Ehlers M.D. (2005). Ubiquitin and protein turnover in synapse function. Neuron.

[61] Vogl A.M., Brockmann M.M., Giusti S.A., Maccarrone G., Vercelli C.A., Bauder C.A., Richter J.S., Roselli F., Hafner A.S., Dedic N. (2015). Neddylation inhibition impairs spine development, destabilizes synapses and deteriorates cognition. Nat. Neurosci..

[62] Scudder S.L., Patrick G.N. (2015). Synaptic structure and function are altered by the neddylation inhibitor MLN4924. Mol. Cell Neurosci..

[63] Dalezios Y., Luján R., Shigemoto R., Roberts J.D., Somogyi P. (2002). Enrichment of mGluR7a in the presynaptic active zones of GABAergic and non-GABAergic terminals on interneurons in the rat somatosensory cortex. Cereb. Cortex.

[64] Niswender C.M., Conn P.J. (2010). Metabotropic glutamate receptors: Physiology, pharmacology, and disease. Annu. Rev. Pharmacol. Toxicol..

[65] Shigemoto R., Kulik A., Roberts J.D.B., Ohishi H., Nusser Z., Kaneko T., Somogyi P. (1996). Target-cell-specific concentration of a metabotropic glutamate receptor in the presynaptic active zone. Nature.

[66] Brockmann M.M., Döngi M., Einsfelder U., Körber N., Refojo D., Stein V. (2019). Neddylation regulates excitatory synaptic transmission and plasticity. Sci. Rep..

[67] Biessels G.J., Reagan L.P. (2015). Hippocampal insulin resistance and cognitive dysfunction. Nat. Rev. Neurosci..

[68] Guillemot-Legris O., Muccioli G.G. (2017). Obesity-Induced Neuroinflammation: Beyond the Hypothalamus. Trends Neurosci..

[69] De Felice F.G., Lourenco M.V., Ferreira S.T. (2014). How does brain insulin resistance develop in Alzheimer’s disease?. Alzheimers Dement..

[70] Lyra E.S.N.M., Gonçalves R.A., Boehnke S.E., Forny-Germano L., Munoz D.P., De Felice F.G. (2019). Understanding the link between insulin resistance and Alzheimer’s disease: Insights from animal models. Exp. Neurol..

[71] Confettura A.D., Cuboni E., Ammar M.R., Jia S., Gomes G.M., Yuanxiang P., Raman R., Li T., Grochowska K.M., Ahrends R. (2022). Neddylation-dependent protein degradation is a nexus between synaptic insulin resistance, neuroinflammation and Alzheimer’s disease. Transl. Neurodegener..

[72] Radhakrishnan D.M., Goyal V. (2018). Parkinson’s disease: A review. Neurol. India.

[73] Xiong H., Wang D., Chen L., Choo Y.S., Ma H., Tang C., Xia K., Jiang W., Ronai Z., Zhuang X. (2009). Parkin, PINK1, and DJ-1 form a ubiquitin E3 ligase complex promoting unfolded protein degradation. J. Clin. Investig..

[74] Um J.W., Han K.A., Im E., Oh Y., Lee K., Chung K.C. (2012). Neddylation positively regulates the ubiquitin E3 ligase activity of parkin. J. Neurosci. Res..

[75] Tanji K., Tanaka T., Mori F., Kito K., Takahashi H., Wakabayashi K., Kamitani T. (2006). NUB1 suppresses the formation of Lewy body-like inclusions by proteasomal degradation of synphilin-1. Am. J. Pathol..

[76] Tanji K., Miki Y., Mori F., Kon T., Kakita A., Takahashi H., Wakabayashi K. (2019). Phosphorylated NUB1 distinguishes α-synuclein in Lewy bodies from that in glial cytoplasmic inclusions in multiple system atrophy. Brain Pathol..

[77] Walton C., King R., Rechtman L., Kaye W., Leray E., Marrie R.A., Robertson N., La Rocca N., Uitdehaag B., van der Mei I. (2020). Rising prevalence of multiple sclerosis worldwide: Insights from the Atlas of MS, third edition. Mult. Scler..

[78] Hauser S.L., Oksenberg J.R. (2006). The neurobiology of multiple sclerosis: Genes, inflammation, and neurodegeneration. Neuron.

[79] Baecher-Allan C., Kaskow B.J., Weiner H.L. (2018). Multiple Sclerosis: Mechanisms and Immunotherapy. Neuron.

[80] Czuczman N.M., Barth M.J., Gu J., Neppalli V., Mavis C., Frys S.E., Hu Q., Liu S., Klener P., Vockova P. (2016). Pevonedistat, a NEDD8-activating enzyme inhibitor, is active in mantle cell lymphoma and enhances rituximab activity in vivo. Blood.

[81] Giordana M.T., Richiardi P., Trevisan E., Boghi A., Palmucci L. (2002). Abnormal ubiquitination of axons in normally myelinated white matter in multiple sclerosis brain. Neuropathol. Appl. Neurobiol..

[82] Minagar A., Ma W., Zhang X., Wang X., Zhang K., Alexander J.S., Gonzalez-Toledo E., Albitar M. (2012). Plasma ubiquitin-proteasome system profile in patients with multiple sclerosis: Correlation with clinical features, neuroimaging, and treatment with interferon-beta-1b. Neurol. Res..

[83] Belogurov A., Kudriaeva A., Kuzina E., Smirnov I., Bobik T., Ponomarenko N., Kravtsova-Ivantsiv Y., Ciechanover A., Gabibov A. (2014). Multiple sclerosis autoantigen myelin basic protein escapes control by ubiquitination during proteasomal degradation. J. Biol. Chem..

[84] Kim K., Pröbstel A.K., Baumann R., Dyckow J., Landefeld J., Kogl E., Madireddy L., Loudermilk R., Eggers E.L., Singh S. (2021). Cell type-specific transcriptomics identifies neddylation as a novel therapeutic target in multiple sclerosis. Brain.

[85] Ross C.A., Tabrizi S.J. (2011). Huntington’s disease: From molecular pathogenesis to clinical treatment. Lancet Neurol..

[86] Kaltenbach L.S., Romero E., Becklin R.R., Chettier R., Bell R., Phansalkar A., Strand A., Torcassi C., Savage J., Hurlburt A. (2007). Huntingtin interacting proteins are genetic modifiers of neurodegeneration. PLoS Genet..

[87] Yao Y., Lu B. (2015). NUB1 suppression of Huntington toxicity: Mechanistic insights. Res. Rep. Biochem..

[88] Lu B., Al-Ramahi I., Valencia A., Wang Q., Berenshteyn F., Yang H., Gallego-Flores T., Ichcho S., Lacoste A., Hild M. (2013). Identification of NUB1 as a suppressor of mutant Huntington toxicity via enhanced protein clearance. Nat. Neurosci..

[89] Lin Y., Xue J., Deng J., He H., Luo S., Chen J., Li J., Yu L., Zhao J., Chen J. (2020). Neddylation activity modulates the neurodegeneration associated with fragile X associated tremor/ataxia syndrome (FXTAS) through regulating Sima. Neurobiol. Disease.

[90] Paulson H., Shakkottai V., Adam M.P., Everman D.B., Mirzaa G.M., Pagon R.A., Wallace S.E., Bean L.J.H., Gripp K.W. (1993–2022). Spinocerebellar Ataxia Type 3. 1998 Oct 10 [Updated 2020 Jun 4]. GeneReviews^®^ [Internet].

[91] Ferro A., Carvalho A.L., Teixeira-Castro A., Almeida C., Tomé R.J., Cortes L., Rodrigues A.-J., Logarinho E., Sequeiros J., Macedo-Ribeiro S. (2007). NEDD8: A new ataxin-3 interactor. Biochim. et Biophys. Acta (BBA)—Mol. Cell Res..

